# A Novel and Selective Poly (ADP-Ribose) Polymerase Inhibitor Ameliorates Chemotherapy-Induced Painful Neuropathy

**DOI:** 10.1371/journal.pone.0054161

**Published:** 2013-01-10

**Authors:** Lauren E. Ta, James D. Schmelzer, Allan J. Bieber, Charles L. Loprinzi, Gary C. Sieck, Jill D. Brederson, Philip A. Low, Anthony J. Windebank

**Affiliations:** 1 Department of Neurology, Mayo Clinic, College of Medicine, Rochester, Minnesota, United States of America; 2 Department of Neuroscience, Mayo Clinic, College of Medicine, Rochester, Minnesota, United States of America; 3 Program in Molecular Neuroscience, Mayo Clinic, College of Medicine, Rochester, Minnesota, United States of America; 4 Division of Medical Oncology, Mayo Clinic, College of Medicine, Rochester, Minnesota, United States of America; 5 Department of Physiology and Biomedical Engineering, Mayo Clinic, College of Medicine, Rochester, Minnesota, United States of America; 6 Neuroscience Research, Global Pharmaceutical Research and Development, Abbott Laboratories, Abbott Park, Illinois, United States of America; Robert Wood Johnson Medical School, United States of America

## Abstract

**Background:**

Chemotherapy-induced neuropathy is the principle dose limiting factor requiring discontinuation of many chemotherapeutic agents, including cisplatin and oxaliplatin. About 30 to 40% of patients receiving chemotherapy develop pain and sensory changes. Given that poly (ADP-ribose) polymerase (PARP) inhibition has been shown to provide neuroprotection, the current study was developed to test whether the novel PARP inhibitor compound 4a (analog of ABT-888) would attenuate pain in cisplatin and oxaliplatin-induced neuropathy in mice.

**Results:**

An established chemotherapy-induced painful neuropathy model of two weekly cycles of 10 intraperitoneal (i.p.) injections separated by 5 days rest was used to examine the therapeutic potential of the PARP inhibitor compound 4a. Behavioral testing using von Frey, paw radiant heat, cold plate, and exploratory behaviors were taken at baseline, and followed by testing at 3, 6, and 8 weeks from the beginning of drug treatment.

**Conclusion:**

Cisplatin-treated mice developed heat hyperalgesia and mechanical allodynia while oxaliplatin-treated mice exhibited cold hyperalgesia and mechanical allodynia. Co-administration of 50 mg/kg or 25 mg/kg compound 4a with platinum regimen, attenuated cisplatin-induced heat hyperalgesia and mechanical allodynia in a dose dependent manner. Similarly, co-administration of 50 mg/kg compound 4a attenuated oxaliplatin-induced cold hyperalgesia and mechanical allodynia. These data indicate that administration of a novel PARP inhibitor may have important applications as a therapeutic agent for human chemotherapy-induced painful neuropathy.

## Introduction

Peripheral neuropathy remains the principle dose limiting toxicity for many chemotherapeutic agents, including plant alkaloids, taxanes, and platinum-based compounds (e.g., cisplatin, oxaliplatin, and carboplatin) [1]. Cisplatin has been used for 40 years to treat many cancers, especially testicular, ovarian, and bladder cancers [2], [3]. Oxaliplatin is the third-generation platinum analogue that has been effective for treatment of colorectal cancer [4], [5], [6].

The severity of neuropathy correlates to the cumulative dose of chemotherapeutic agent administered. Cisplatin, for example, induces axonal neuropathy, characterized by a glove-and stocking distribution of sensory changes including dysesthesia, paraesthesia, and pain, at a cumulative dose >400–500 mg/m^2^. These symptoms frequently develop several weeks to months after initiation of chemotherapy and improve after treatment ends [7]. However, for some patients, these symptoms can progress for weeks after therapy is discontinued and may result in numbness, tingling, and chronic pain [1].

Oxaliplatin induces two different forms of neuropathy [8], [9]. A dose-limiting sensory neuropathy that occurs at a cumulative dose >540–850 mg/m^2^ may develop gradually after oxaliplatin treatment, similar to that seen with cisplatin [10]. In addition, a transient, acute syndrome consisting of cold-induced paraesthesia, dysesthesia or pain in the hands, feet, face, and perioral regions appears in 80% of patients during the first or second cycle [10]. Current therapeutic options for neuropathy are largely limited to drugs approved for other pain conditions; none of them have been proven to provide relief [11], [12]. As chemotherapy becomes more successful, the number of cancer survivors will likely increase and neuropathy will become an important factor limiting quality of life.

Platinum drugs target tumor cells by forming DNA-platinum adducts that stall replication machinery and trigger apoptotic signaling cascades [13]. However, in the case of post mitotic neurons, the cause of cytotoxicity is less clear. We have shown that dorsal root ganglion (DRG) neurons accumulate high levels of platinum–DNA adducts in a time-dependent manner following cisplatin- and oxaliplatin exposure [14], [15]. This platinum binding has been shown to induce neurons to enter the cell cycle and undergo apoptosis [16]. Similarly, neuronal survival correlates with DNA repair capacity [17], [18]. Peripheral sensory neurons are particularly vulnerable to platinum drugs because they are not protected by the blood brain barrier. It remains unclear whether these events are the primary factors that trigger a cascade of events leading to neuronal damage that contributes to neuropathic pain.

A number of endogenous cellular repair mechanisms, including the nucleotide-excision repair (NER), base excision repair (BER), and double strand break repair (DSBR) pathways, can remove platinum-DNA lesions thereby reinstating replication, blocking apoptosis, and limiting the efficacy of chemotherapy [19], [20]. Activity of these pathways is often increased in cancer cells, creating chemotherapy resistance [21]. Modulating repair pathways, therefore, has become a major target for improved chemotherapy [22].

Poly (ADP-ribose) polymerase (PARP) is a family of DNA sensing enzymes that recognize single-stranded DNA strand breaks and initiate repair via the BER pathway. The most abundant member, PARP-1, binds DNA and catalyzes the formation of poly ADP-ribose (PAR) which serves as a docking signal to other DNA repair enzymes such as DNA ligase III (LigIII) and DNA polymerase beta (polβ) [23]. Due to redundancy in DNA repair mechanisms, PARP inhibition may not have a significant effect in normal cells or some types of cancer. However, in select tumors with a BRCA mutation that lack homologous recombination, PARP inhibition triggers cell death via a synthetic lethality process [24]. Since NAD+ acts as the substrate for generation of ADP-ribose monomers, persistent activation of PARP can deplete the cell of ATP, perturbing cellular homeostasis and triggering cell death. Furthermore, activation of PARP has been shown to initiate various apoptotic signaling events [23]. Therefore, depending on the context and extent of PARP activation, it can either be beneficial or detrimental to cellular homeostasis.

PARP inhibition has been successfully employed as a novel therapeutic strategy in cancer therapy to enhance the cytotoxic effects of DNA-damaging agents [24]. ABT-888 (veliparib) is a novel and potent PARP-1 and PARP-2 inhibitor that has been shown to potentiate multiple DNA damaging agents including cisplatin, carboplatin, cyclophosphamide, and temozolomide [25] and has currently progressed into human phase II clinical trials. Moreover, findings from a phase I clinical trial demonstrated that olaparib, another PARP inhibitor, increases antitumor efficacy of traditional chemotherapy with less adverse effects [26]. While PARP inhibition has shown therapeutic benefit in cancer treatment, its application has also improved the outcome in a variety of neuropathological conditions including a model of diabetes [27]. Compound 4a is the enantiomer of the clinical PARP inhibitor veliparib (ABT-888) and is a potent inhibitor of both PARP-1 and 2 (K_i_ of 5 and 2 nM for PARP-1 and PARP-2, respectively) and has an EC_50_ of 3 nM in a cell based assay of PARP activity [28]. The mouse pharmacokinetic profile of compound 4a is very similar to ABT-888 and demonstrates equivalent *in vivo* efficacy in mouse xenograft tumor models. In the current investigation, we hypothesized that the selective PARP inhibitor compound 4a would attenuate cisplatin and oxaliplatin-associated pain.

## Materials and Methods

### Experimental Animals

Male C57BL6J mice were acclimated to their enriched environment in cages of four for 2–4 weeks prior to study. Mice were 10–12 weeks old and weighed 24–26 g when the study began. All mice had free access to water and food and were exposed to a standard light cycle of 12 hours on and 12 hours off. This study was conducted with the approval of the Mayo Clinic Animal Care and Use Committee, in compliance with the regulations of the National Institutes of Health and the ethical guidelines of the International Association for the Study of Pain [29].

### Cisplatin and Oxaliplatin-Induced Painful Neuropathy Models

Cisplatin and oxaliplatin-induced painful neuropathy models were performed as previously described [30], [31]. Oxaliplatin (Sigma-Aldrich, St. Louis, MO) was dissolved in 5% dextrose (1 mg/ml) and prepared fresh for daily use. Pharmaceutical grade cisplatin (1 mg/ml) was obtained from Bristol-Myers Squibb Company (Princeton, NJ) in 0.9% saline. After habituation to the test environment and baseline measurements of pain sensitivity, mice were randomized to three treatment groups of either cisplatin (2.3 mg/kg), oxaliplatin (3.0 mg/kg), or vehicle (0.9% saline). Using injection volume of 10 ml/kg, mice were treated with daily intraperitoneal (i.p.) administration for 5 days, followed by 5 days of rest, for two weekly cycles. Total cumulative doses of 23 mg/kg cisplatin and 30 mg/kg oxaliplatin over a total of ten injections were used.

### PARP Inhibitor (compound 4a)

**Figure 1:**
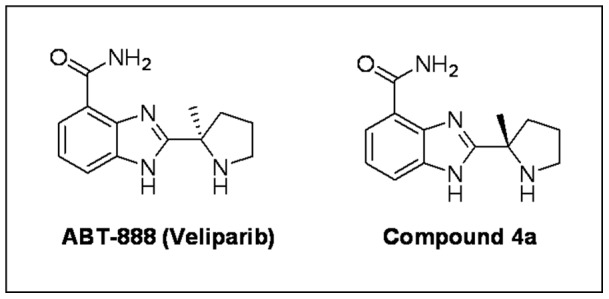
Chemical structure of ABT-888 and compound 4a (C_13_H_16_N_4_O, 244.29 g/mol).

Compound 4a (Fig. 1) was provided by Abbott Laboratories (Abbott Laboratories, Abbott Park, IL). Doses of 50 mg/kg or 25 mg/kg in PBS solution were administered by i.p. injection two days prior to treatment with cisplatin or oxaliplatin, with administration continuing by i.p. injection along with the cisplatin or oxaliplatin regimen.

### Behavioral Studies

After baseline measurements of pain sensitivity, mice were randomized into 7 experimental groups: cisplatin, oxaliplatin, vehicle, cisplatin+compound 4a (50 mg/kg), cisplatin+compound 4a (25 mg/kg), oxaliplatin+compound 4a (50 mg/kg) and compound 4a (50 mg/kg) alone. All behavioral tests were conducted at room temperature (25°C) and between the hours of 0800 and 1600 by an operator who was blinded to the drug treatment condition. All behavioral assays (locomotor activity, von Frey, radiant heat, and cold plate) were conducted as previously described [30], [31].

#### Activity Assay

Monitoring of locomotor activity was measured using VersaMax Animal Activity Monitors (AccuScan Model RXYZCM-16, Columbus, OH). Total distance traveled in 20 min was analyzed using VersaMax Analyzer (AccuScan Model CDA-8, Columbus, OH). Data were averaged and presented as the mean ± SEM.

#### Cold Plate Assay

Cold hyperalgesia was measured by a Peltier-cooled cold plate (TECA, Chicago, Il) preset at −2.5° ±0.2°C. The number of paw lifts in 5 min was video recorded for counting. Three trials on three separate days were averaged and presented as the mean ± SEM.

#### Radiant Heat Assay

Thermal hyperalgesia was measured in a radiant heat apparatus using a Plantar Ugo Basile (Stoelting, Wood Dale, Il). Paw withdrawal latency from both hind paws was averaged over eight trials and presented as the mean ± SEM.

#### Von Frey Assay

Mechanical allodynia was assessed by an Ugo Basile Dynamic Plantar Aesthesiometer (Stoelting, Wood Dale, Il) using the von Frey filament principle. Paw withdrawal threshold from both hind paws was averaged over eight trials and presented as the mean ± SEM.

### Statistical Analyses

Data are expressed as the mean ± SEM. Results were illustrated and analyzed using Graphpad Prism version 4 (Graphpad Software, San Diego, USA). Statistical analyses were performed using two-way ANOVA to examine the differences in response across treatment groups with drug treatments and time as independent variables. Follow-up analysis was conducted using the Bonferroni test. *P*<0.05 was considered statistically significant.

## Results

### Evaluation of General Toxicity and Activity Assay

In order to monitor well-being and determine drug dosing, mice were observed and weighed daily throughout the experiment. All mice survived until the end of study. There were no signs of distress, deterioration of general health, or evidence of severe general toxicity. Repeated treatment to cumulative doses of 23 mg/kg cisplatin and 30 mg/kg oxaliplatin have been shown to induce neuropathy associated behavioral hypersensitivity in mice without causing renal damage (BUN level >40 mg/dL) or severe body weight loss (>20%), which would require euthanasia [30], [31].

### PARP Inhibitor Compound 4a Treatment does not Attenuate Cisplatin and Oxaliplatin-induced Body Weight Loss

To examine the effects of PARP inhibitor compound 4a on reduction of body weight in the chemotherapy-induced neuropathy model, mean time course body weight was evaluated in mice after platinum treatment with or without PARP inhibitor.

**Figure 2:**
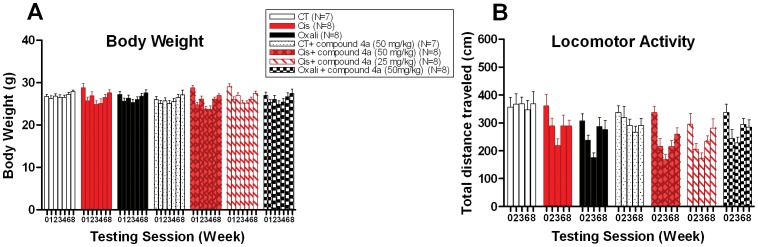
PARP inhibitor compound 4a does not affect the decline in body weight and exploratory behavior associated with cisplatin and oxaliplatin-induced neuropathy. **(A)**. Time course of mice mean body weight is shown after i.p. administration of 50 mg/kg compound 4a in combination with platinum drugs, platinum drugs alone, or vehicle. **(B)** Time course of mean horizontal distance traveled in 20 min is shown after i.p. dosing with 50 mg/kg compound 4a in combination with platinum drugs, platinum drugs alone, or vehicle. Similarly, no effect is observed with 50 mg/kg compound 4a on control mice. Data represent the mean ± S.E.M. of 7–8 mice, two-way ANOVA followed with post hoc analysis.

The dosing regimen was well tolerated with combination regimens (Fig. 2A). Indeed, after two cycles of dosing, 50 m/kg compound 4a or 25 mg/kg compound 4a with cisplatin combination showed no significant difference in mean body weight compared to cisplatin alone (Fig. 2A). The mean body weight loss was 17% for the 50 mg/kg compound 4a and cisplatin combination compared with 13% for 25 mg/kg compound 4a and cisplatin combination, and 14% for cisplatin alone (Fig. 2A).

Conversely, only 8% of mean body weight loss was seen for the 50 mg/kg compound 4a and oxaliplatin combination compared with 7% for oxaliplatin alone (Fig. 2A). All mice gradually return to their basal body weight level after drug cessation (Fig. 2A).

### PARP Inhibitor Compound 4a does not Affect the Decline in Exploratory Behavior Associated with Cisplatin and Oxaliplatin Treatment

Platinum-drug induced neurotoxicity has been shown to associate with decline in exploratory behaviors in mice [30], [31]. To evaluate whether PARP inhibitor compound 4a alters the exploratory activity, we monitored mouse locomotor activity in an open field by measuring the horizontal distance traveled after platinum treatment with or without PARP inhibitor.

No significant changes in exploratory behavior were observed when a combination regimen was used (Fig. 2B). Dosing at 50 m/kg compound 4a or 25 mg/kg compound 4a in combination with cisplatin, showed no significant difference in exploratory behaviors compared to cisplatin alone (Fig. 2B). The mean decline in horizontal distance traveled was 50% for the 50 mg/kg compound 4a and cisplatin combination compared to 41% for 25 mg/kg compound 4a and cisplatin combination, and 40% for cisplatin alone (Fig. 2B).

Similarly, dosing at 50 m/kg compound 4a in combination with oxaliplatin, showed no significant difference in exploratory behaviors compared to oxaliplatin alone (Fig. 2B). The mean reduction in horizontal distance traveled was 32% for the 50 mg/kg compound 4a and oxaliplatin combination compared with 43% for oxaliplatin alone (Fig. 2B). All mice gradually return to their basal exploratory activity after drug cessation (Fig. 2B).

### PARP Inhibitor Compound 4a Attenuates Mechanical Allodynia in Cisplatin and Oxaliplatin-induced Neuropathy

To determine whether PARP inhibitor could reduce the development of mechanical hypersensitivity behavior in chemotherapy-induced neuropathy, we measured paw withdrawal thresholds in mice after platinum treatment with or without PARP inhibitor.

**Figure 3:**
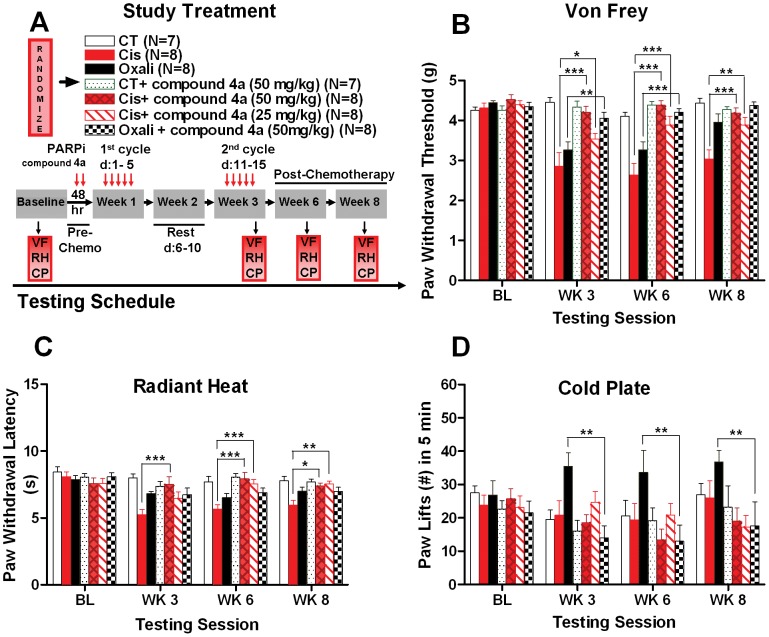
PARP inhibitor compound 4a attentuates mechanical allodynia, thermal hyperalgesia, and cold hyperalgesia associated with cisplatin and oxaliplatin-induced neuropathy. **(A)** After baseline behavioral testing, mice are randomized to experimental groups and PARP inhibitor compound 4a treatment by i.p. injection started two days before and continued with cisplatin or oxaliplatin drug regimen. Behavioral testing were conducted 24 hours after the last 10^th^ doses at week 3, and at post- chemotherapy or follow up evaluation at weeks 6 and 8. Behavioral assays: VF (VonFrey), RH (Radiant Heat), CP (Cold Plate). **(B)** Dosing with 50 mg/kg compound 4a or 25 mg/kg compound 4a in combination with cisplatin reversibly reduces mechanical allodynia in cisplatin- induced neuropathy mice compared to cisplatin alone at weeks 3, 6, and 8. Similarly, i.p. dosing with 50 mg/kg compound 4a in combination with oxaliplatin prevents mechanical allodynia in oxaliplatin-induced neuropathy mice compared to oxaliplatin alone at weeks 3 and 6. **(C)**. Dosing with 50 mg/kg of compound 4a in combination with cisplatin improves thermal hyperalgesia in cisplatin-induced neuropathy mice compared to cisplatin alone at weeks 3, 6, and 8. Similarly, dosing with 25 mg of compound 4a in combination with cisplatin reduces thermal hyperalgesia in cisplatin-induced neuropathy mice compared to cisplatin alone at weeks 6 and 8. Oxaliplatin did not elicit thermal hyperalgesia in mice. **(D)**. Dosing with 50 mg/kg compound 4a in combination with oxaliplatin reduces oxaliplatin-induced cold hyperalgesia compared to oxaliplatin alone at weeks 3, 6, and 8. Cisplatin did not induce cold hyperalgesia in mice. No effect of compound 4a on control mice. Data represent the mean ± S.E.M. of 7–8 mice, *P<0.05; **P<0.01; ***P<0.001, two-way ANOVA followed with post hoc analysis.

Platinum drugs induced mechanical hypersensitivity with different temporal profiles (Fig. 3B). Behavioral signs of mechanical allodynia were evident after two cycles (week 3) of dosing with cisplatin or oxaliplatin. While oxaliplatin only elicited pain behavior through week 6, cisplatin-induced behaviors persisted through week 8 (Fig. 3B).

PARP inhibitor compound 4a inhibited mechanical allodynia in a dose dependent manner. Co-administration of 50 mg/kg compound 4a with cisplatin significantly increased mean withdrawal thresholds by 47%, 66%, and 38% compared to cisplatin alone at weeks 3, 6, and 8, respectively (Fig. 3B). Compound 4a dosed at 25 mg/kg with cisplatin was not as effective but resulted in significantly increased mean withdrawal thresholds of 24%, 48%, and 28% compared to cisplatin alone at weeks 3, 6, and 8, respectively (Fig. 3B).

Combination of 50 mg/kg compound 4a with oxaliplatin also effectively reduced mechanical allodynia, resulting in 24% and 29% increases in mean withdrawal thresholds compared to oxaliplatin alone at weeks 3 and 6, respectively (Fig. 3B).

### PARP Inhibitor Compound 4a Attenuates Thermal Hyperalgesia in Cisplatin-Induced Neuropathy

To investigate the therapeutic potential of PARP inhibitor in preventing the development of heat hypersensitivity in chemotherapy-induced neuropathy, we measured paw withdrawal latency in response to radiant heat.

Consistent with our previous findings [30], [31], cisplatin- but not oxaliplatin-treated mice displayed enhanced sensitivity to noxious heat as measured by radiant heat-evoked withdrawal latency (Fig. 3C).

In combination with cisplatin, PARP inhibitor compound 4a attenuated thermal hyperalgesia in a dose-dependent fashion (Fig. 3C). Compound 4a dosed at 50 mg/kg with cisplatin, resulted in significant increases of 44%, 40%, and 24% in mean radiant heat evoked withdrawal latency compared to cisplatin alone at weeks 3, 6, and 8, respectively (Fig. 3C). Compound 4a dosed at 25 mg/kg with cisplatin also significantly reduced thermal hyperalgesia but only at weeks 6 (33% increase compared to cisplatin alone) and 8 (27% increase compared to cisplatin alone) (Fig. 3C).

### PARP Inhibitor Compound 4a Attenuates Cold Hyperalgesia Associated with Oxaliplatin-induced Neuropathy

To test whether the application of PARP inhibitor reduces the development of oxaliplatin- induced cold hyperalgesia, cold pain behavioral responses were measured in mice after platinum treatment with or without PARP inhibitor**.**

Oxaliplatin, but not cisplatin, induced cold hyperalgesia, was measured as the number of paw lifts at −2.5°C (Fig. 3D). This cold-evoked hyperalgesia was prevented by combined treatment with compound 4a. At 50 mg/kg, compound 4a significantly reduced the mean number of paw lifts by 60%, 61%, and 52% compared to oxaliplatin alone, at weeks 3, 6, and 8, respectively (Fig. 3D).

## Discussion

Cancer related pain such as chemotherapy-induced painful neuropathy is a major morbidity caused by many commonly used chemotherapeutic agents for cancer therapy. Pain can be disabling, causing loss of functional abilities and decreased quality of life. Current therapeutic options for CIPN are largely limited to drugs approved for other pain conditions such as anticonvulsants, antidepressants, and opioids, which offer minimal relief [11], [12]. The clinically relevant goal of this study was to test whether a novel PARP inhibitor, compound 4a, can attenuate chemotherapy-induced neuropathic pain.

Chemotherapy treatment with cisplatin or oxaliplatin produces painful neuropathy characterized by reduced thresholds to mechanical stimuli. Cisplatin reduced thresholds to heat, and in contrast, oxaliplatin reduced thresholds to cold stimuli [30], [31]. In this study we demonstrate that the novel and selective PARP-1/2 inhibitor, compound 4a, provides a protective effect against the functional sensory deficits, as measured by behavioral parameters induced by cisplatin and oxaliplatin treatment.

The platinum drug doses used in this study are based on therapeutic doses that induce neuropathy in humans [7], [8], [9], [10] and mice [30], [31] and have antitumor activity [25], [32] in mice. As in humans, the cumulative dose and time course of administration predicts severity of associated sensory deficits in rodent studies after cisplatin [33], [34], [35], [36] and oxaliplatin treatment [37], [38], [39], [40], [41], [42]. The cisplatin dose at 2.3 mg/kg, given in 10 doses in a 3 weeks regimen, was calculated as a dose equivalent of 80 mg/m^2^ of body surface area, resulting in a total dose of 800 mg/m^2^. Similarly, oxaliplatin at 3.0 mg/kg given in 10 doses is equivalent to 105 mg/m^2^ of body surface area with a total dose of 1050 mg/m^2^.

Both cisplatin and oxaliplatin induced neuropathy in mice was characterized by a robust behavioral hypersensitivity to mechanical stimuli. While cisplatin-treated mice developed heat hyperalgesia, oxaliplatin induced a cold-sensitive neuropathy, consistent with previous reports [30], [31], [41]. Compared to oxaliplatin, cisplatin induced more severe neuropathy since symptoms persisted for weeks after cessation of oxaliplatin-induced symptoms. This is consistent with our previous in vivo studies [30], [31] and our in vitro observations of neurotoxicity [15].

The present study shows that i.p. co-administration of compound 4a with platinum drugs provides dose-dependent antinociceptive efficacy in mechanical, heat, and cold assays. Cisplatin-induced heat hyperalgesia and mechanical allodynia were significantly reduced following co-administration of compound 4a at either 25 or 50 mg/kg. Similarly, cold hyperalgesia and mechanical allodynia resulting from treatment with oxaliplatin were reduced by co-administration of compound 4a at 50 mg/kg. Using parallel group comparison in a single mouse model, these findings provide the first evidence that a novel PARP inhibitor compound 4a reduce pain in mice associated with both cisplatin and oxaliplatin treatment.

Nerve injury induced pain results from activation of small-fibers: Aδ and C-fibre polymodal nociceptors. These nociceptors can be sensitized by various ion channels and inflammatory mediators whose sustained presence may lead to peripheral or central sensitization manifesting as prolonged neuronal discharges, response to non-noxious stimuli, amplified responses to noxious stimuli, and expansion of the receptive field. Continuous and intense nociceptive barrage from primary afferents may drive central sensitization, which most likely also plays an important role in human chronic pain [43], [44]. Such activation and sensitization of nociceptors and wide dynamic range neurons may explain the observed pathological pain behaviors following platinum drugs [39], [41], [45], [46]. Ion channels, including transient receptor potential (TRP) receptors, contribute to the sensitization of nociceptors following platinum drugs. We have previously demonstrated that TRP vanilloid 1 receptor is required for thermal hyperalgesia in cisplatin-induced neuropathy [31]. Cisplatin and oxaliplatin-induced neuropathy mice exhibit robust behavioral hypersensitivity to mechanical, cold, and heat stimuli. While the causes of these pain behavioral phenotypes are undoubtedly complex, sensitization of the peripheral and central mechanisms in chemotherapy-induced neuropathy likely contributes to the pain patterns.

We have shown that PARP inhibition ameliorates the painful side effects of platinum drugs. This is in line with a recent report in which dosing with PARP inhibitor reduced neuropathic pain behaviors in a rat model of diabetic peripheral neuropathy [27]. Although the mechanism is unclear, it has been demonstrated that activation of PARP in glial cells contributes to neuroinflammation and increased production of the calcium-insensitive nitric oxide synthetase, iNOS [47]. Nitric oxide (NO), as well as other inflammatory byproducts, are capable of directly activating neuronal transient receptor potential channel A1 (TRPA1) [48] which we have previously shown to be up-regulated following platinum treatment in rat dorsal root ganglion *in vitro* and mouse trigeminal ganglion *in vivo* [31]. Increased expression of TRPA1 transcript was also observed after a single dose treatment with oxaliplatin in mouse dorsal root ganglia *in vivo* [49]. TRPA1 is also activated by reactive oxygen species (ROS) and products of oxidative stress-induced lipid peroxidation [50]. Although PARP activity is generally thought to occur as the result of oxidative stress induced DNA damage, several studies using cultured cells have demonstrated that PARP activity can lead to oxidative stress [51], [52], [53]. This suggests that activation of PARP by platinum compounds may result in activation of primary afferent nociceptors through a TRPA1 dependent mechanism. TRPA1 expression is increased in trigeminal ganglion in both cisplatin and oxaliplatin treated mice [31], however, the pain induced by these treatments is qualitatively different. The reason for this remains unclear.

Thus, PARP inhibitors, a new class of agents with potential to improve efficacy of several commonly used chemotherapeutics in oncology, also appear to ameliorate sensory neuropathy in mice. This suggests that neuroprotection through PARP inhibition might have therapeutic benefit for platinum-induced neuropathy in humans. Neuronal DNA damage might be expected following chemotherapy, resulting in extensive PARP-1 activation leading to neuronal dysfunction, aberrant somatosensory processing of the peripheral and/or central nervous system, and subsequent neuropathy. Neuroprotection resulting from inhibition of PARP activity might therefore explain, in part, the mechanistic basis for the observed amelioration in neuropathic pain. Alternatively, PARP inhibition may exacerbate the neurotoxic effects of platinum drug treatment leading to greater peripheral nerve dysfunction. This might manifest as hypoalgesic responses to sensory testing. This scenario may have occurred with the paw lift responses observed in the cold plate assay where co-administration of compound 4a with oxaliplatin appeared to reduce the number of paw lifts compared to the controls at week 3, 6, and 8 (Fig. 3D). However, since the change observed in the number of paw lifts between oxaliplatin with compound 4a compared to controls, was not found to be statistically significant across all time points (P>0.05), this alternative hypothesis could not be considered. Furthermore, combination of oxaliplatin or cisplatin with compound 4a showed a return of sensory responses to baseline levels in both mechanical and heat assays (Fig, 3B and C). It is also an unlikely premise in nociception that a drug which exhibits neuroprotective effects in one pain modality will elicit neurotoxic effects in a different pain modality. Understanding the precise mechanisms that drive persistent painful neuropathy may provide novel mechanism-based therapies to treat these debilitating diseases.

Compound 4a was recently developed as a potent inhibitor of PARP. The compound has good oral bioavailability, can cross the blood-brain barrier, and potentiates the effects of treatment with several chemotherapeutic agents. The properties of this compound make it an attractive candidate for clinical evaluation for chemotherapy-induced painful neuropathy.
